# Cholera

**Published:** 1851-04

**Authors:** T. Y. Chaufa Mongkut


*Philadelphia, Feb. 24th,* 1851.

To the Editors of the Medical Examiner.

GENTLEMEN,—I enclose herewith for your valuable journal an account of Cholera as it appeared in the kingdom of Siam during the year 1849, by His Royal Highness T. Y. Chaufa Mongkut.

It is an extract from the "Supplement to the Bankok Calendar, being a series of communications from His Royal Highness, T. Y. Chaufa Mongkut," printed at Bankok 1850, for a copy of which I am indebted to the politeness of Dr. Hopkinson, U. S. Navy, who has very recently returned from a cruise in the East Indies.

His Royal Highness, Chaufa Mongkut, wears the yellow robe of the Talapoins, and holds the office of High Priest of the Budhist religion. He declined his right to the throne of Siam, in favor of a younger brother, Momfanoi. He speaks English perfectly; the following communication is an evidence of his ability to write it.

According to the government census of 1828, the population of Bankok was 401,300 souls, of which number 360,000 are Chinese or their descendants.

I am, very respectfully, &c.,

W. S. W. RUSCHENBERGER.

*Cholera. Communicated by His Royal Highness* T. Y. CHAUFA MONGKUT.

The Cholera made its appearance first at Pinang in April last year. Vegetation decayed and turned yellow; and after about a fortnight, the Cholera commenced its ravages, many persons dying in four or five hours after its first symptoms appeared. It prevailed about three weeks, and then gradually diminished and seemed to remove to the Malay Coast east of Pinang. It spread into 15 Malay villages which are tributary to Siam, viz.—Kedah, &c. Then into the southern Provinces of Siam, as Patani, Songkhla, Patalung, thence passing up the coast on the west side of the Gulf of Siam, reached Chiyah or Bhumriang early in June. Thence it seemed to pass directly to the mouth of the River at Paknam, not affecting the towns on the Tachin or Meklong rivers, or the villages, Banglem and Bangtabhun. It prevailed at Paknam and vicinity, about June 11th, and passing along both banks of the river, appeard at Bangkok on the 15th, very rife on the 17th and 18th, and such was its violence and fatality, that 2 and 300 inhabitants were swept off per day. It continued to increase till on the 23rd, when Chau Phya Bodin Decha, the celebrated Commander-in-Chief of the Siamese army, fell its victim, together with more than 500 persons residing within the walls of the city. From the 23rd, the violence of the disease somewhat abated, and the number of deaths diminished gradually from day to day, until July 16th, at which date things began to assume an appearance of usual tranquillity.

His Royal Highness, T. Y. Chaufa Mongkut gave direction to his servants to visit three of the principal burning or burial places about Bangkok, viz., Wat Saket, Wat Tinlen, and Wat Banglamphu, to visit them daily and take correct account of all the corpses of persons dying of Cholera—brought there to be disposed of by burning or burying. This was done every day for four weeks, commencing June 17th, to July 15th, and the following table shows the number of dead bodies carried to these three Wats or Temples in Bangkok during two weeks in June and two in July.

| | Wat Saket. | Wat Tinlen. | Wat Banglamphu. | Total. |
|---|---|---|---|---|
| June 17 | 15 | 12 | 8 | 35 |
| 18 | 45 | 57 | 38 | 140 |
| 19 | 135 | 60 | 68 | 253 |
| 20 | 189 | 97 | 83 | 359 |
| 21 | 291 | 122 | 80 | 493 |
| 22 | 344 | 190 | 135 | 669 |
| 23 | 365 | 200 | 131 | 696 |

| | | | | |
|---|---|---|---|---|
| 24 | 344 | 150 | 94 | 588 |
| 25 | 226 | 70 | 96 | 382 |
| 26 | 182 | 50 | 75 | 307 |
| 27 | 153 | 52 | 66 | 271 |
| 28 | 86 | 30 | 59 | 185 |
| 29 | 71 | 60 | 50 | 181 |
| 30 | 64 | 25 | 43 | 112 |
| July 1 | 31 | 59 | 41 | 131 |
| 2 | 53 | 41 | 20 | 114 |
| 3 | 26 | 26 | 13 | 65 |
| 4 | 31 | 31 | 12 | 74 |
| 5 | 16 | 26 | 11 | 53 |
| 6 | 21 | 26 | 16 | 63 |
| 7 | 13 | 20 | 14 | 47 |
| 8 | 14 | 10 | 3 | 27 |
| 9 | 11 | 15 | 15 | 41 |
| 10 | 12 | 10 | 16 | 38 |
| 11 | 4 | 10 | 9 | 23 |
| 12 | 7 | 10 | 8 | 25 |
| 13 | 11 | 10 | 5 | 26 |
| 14 | 7 | 12 | 4 | 23 |

Total in 28 days, 5457 ;—average per day, 137. Greatest mortality on the 23rd of June.

From various accounts His R. H. and other considerate persons believe the number of bodies carried to other places from the city and its walls were not more than 1000, besides those above and below—and on the western banks of the river, might be about 6,000 more. This estimate is made by comparing the deaths in various groups of houses extending over limited spaces. It was judged that about one-tenth of the population were swept away by the cholera.

The estimate is placed higher by some ignorant persons, whose fears were unnecessarily awakened, so that they could not give the subject sufficiently careful attention. None, however, doubted but that about 15,000 souls were swept away by this pestilence. Notwithstanding the calamity was so fatal, it fell generally and mostly on prisoners, slaves and common people ; but persons of distinction and noblemen, dwelling in high habitations or in floating houses, where the tide flowed regularly, died only in very small numbers. A few princes and princesses and some noblemen of rank, however, fell victims to the scourge.

But those persons who were absent in China and Batavia, had they returned without having heard the tidings, would scarcely apprehend or perceive that any thing extraordinary had happened, or that there was any observable difference or diminution of the people by the death of one-tenth. His Royal Highness regrets that the Siamese Government could not secure an exact census both of the living and the dead—on account of the excitement that prevailed. He has endeavored to ascertain the number of deaths compared with former attacks of the disease. From Bangkok it spread in every direction. On the west to Tachin, Nakon Chaisi, Suphan, Rajaburi, and Mehlong. On the north—to the farthest provinces; and then down south again to Chumpon and Chai yat on the Gulf formerly mentioned,–and on the east side of the Gulf all along to Kambuja. He has learned that the disease raged most at Ayudia in the early part of July, a few days later than at Bangkok. Here the deaths were rare in houses situated within the fosse of the city and in floating houses, but more numerous on the Ta Khian canal. The disease did not reach Pitsanulok, north-eastern province, till late in August. And in the central northern provinces of Sawankhalok and Sukkothai, and western dirtrict of Kampeng pet—not till early in September. Its appearance in the northern parts of Siam, south of Chiang Mai was late in July and early in August. His Royal Highness requested several officers of various small towns to furnish him accounts of the number of persons who perished by the Cholera, together with general statements in regard to the whole population; and he was assured that about one-tenth died in most of the places; except, Anghin, Bangphra, &c., on the east side of the Gulf down to Chantibun, in which places there were but few deaths. The following are *particular examples.*

At Wat Bawarniwes where His Royal Highness resides, the priests and laymen amount to 396;—26 of them died in June. In some places, the proportion was greater. Samkhok—whole population 1254; died 96. The town of Tha Dhani, province of Sawankalok had a population of 558, died of Cholera, 57. At Kahuyang near Kampeng pet on the N. W., out of a population of 1039, 103 died.

At Anghin, east of the Gulf, with a population of 1,025, only

5 died; while at Blangplasoi, only a few miles distant, the deaths were very numerous, which is a remarkable circumstance. But the former is high land, receding from a muddy shore, and the latter low ground terminating in banks of mud and sand. Anghin is subject to a variety of terrible diseases, as fevers, dysentery, small pox, &c., which occasionally prevail in other parts of the country. It is exposed to all winds but the east.

The appearance of the Cholera was unknown till 1819; it was heard of first in India. Some Siamese from Trang, on the west coast of Siam, witnessed the disease, and immediately returned to Siam, and brought the astonishing tidings before the Cholera reached Siam in 1820. It first broke out in Pinang and Trang and followed the same course as this year, by Chaiyah, and Paknam to Bangkok. Thus in 29 years, from 1820—1849, the Cholera has raged twice with great violence. During other years of this period, it has occasionally appeared for a week, but the cases were few.

His Royal Highness thinks it reasonable to suppose that it originates from the influence of volcanic and poisonous gases, or vapor which would be drawn forth by the attraction of the heavenly bodies in their various conjunctions and oppositions, but almost all the officers of government and the people generally, suppose the Cholera to be an attack of an army of demons, or cruel and malicious spirits, who come invisibly, to seize mankind, and make them their companions or servants, or that this calamity was sent by God, as an expression of his displeasure. His Royal Highness and his associates were greatly displeased with such unreasonable, delusive superstitions, and having no confidence in such views, many have become his enemies and strongly opposed him on this subject. The people generally endeavored to check the spread of the Cholera, and protect themselves and families by offerings and prayers to god, gods, and goddesses and evil spirits, with numerous incredulous and ridiculous ceremonies.

His Royal Highness greatly regrets his past neglect to observe the thermometer and barometer, that he might have noted the state of the temperature, and atmosphere at the time of the first attack of Cholera in 1820. He had these instruments when he was 16 years old, but he had no person to explain their use and importance, and no minutes were made of what they indicated at that time. But he well remembers that the cold was regularly increasing from day to day, from December, through January, and then diminishing till the close of February, when it was very warm. This he recollects as being a uniform process from his youth to the year 1818, when in May, the heat was unusually intense, and in the December and January following, the cold was more severe, but in 1820, before the Cholera commenced, the cold was less, and the heat greater, so that the people said they have never seen the like. In April of that year, the Small Pox raged with fearful violence, and then followed the Cholera in much the same manner as this year. It occurred on the Malay Peninsula in March, when the sun was near the equator, about the vernal equinox, Saturn and Jupiter being in the node, in the signs of Pisces and Aries.

During the 24 years from 1820, whenever the Cholera made its irregular appearance, it has always been when the heat was greater, and the cold diminished or discontinued for many days. Previous to the year 1848, His Royal Highness never observed the thermometer to be above 98° in April, May or June, and its lowest range in winter 60°, and that only for an hour or two in the morning. There was no day when it did not rise to over 70° in the course of the day. The cold has decidedly diminished in Siam since 1818. As evidence of it, we have only to look at the garments of the people. The jackets they used to wear, are scarce and seldom to be seen, almost wholly gone out of use, as this winter '49–50. But in the winter of '48–49, it was cold in November and December, and increased for nearly the two months. Sometimes the thermometer was down to 57° and 58° and continued so for 3 or 4 hours every day in the middle of December, and many successive days, did not exceed 70°. After the first of January, the season soon turned to be hot. This increased till the latter part of April, so that when the sun was in the zenith at Paknam, Bangkok and Ayudia, the temperature was 106°, 105° and 104°, and was not less than 100° till about sunset, and late at night, when it was 98° and 97°. The thermometer shows that the heat was greater in April and May 1849, than for many years before. But when the Cholera appeared in June, the heat began gradually to diminish to 90°, 89°, 88°, and 87°

during the hottest hours of the day, and 81° or 80 in the night; and the barometer showed that the atmosphere was very rare. The difference of similar remarkable situations of planets or heavenly bodies on this and previous occasions of Cholera, was this,—The planet Jupiter had crossed the equinoctial point under the planet Saturn, which was with the node in the same geocentric sign between Pisces and Aries on the last occasion: but on this occasion the planet Mars instead of Jupiter, on the 14th of May last, had crossed the equinoctial point under the planet Saturn, which was on the same place of its former revolution and opposed with the node, which little proceeding from opposition between the signs of Virgo and Libra.

His Royal Highness and his associates have examined whether there were any other such conjunctions, and find none for thirty years or fifteen years past. Hence he concludes that the Cholera has been occasioned by these conjunctions, drawing out the poisonous or deleterious gases, engendered by iron ores and mineral substances, as he has observed that its ravages were greater where such ores and mineral substances abounded, than in sandy and alluvial grounds.

His Royal Highness wished to write also in regard to the curative treatment of Cholera, but regrets he has not the leisure. He does not know how to state many things which would be appropriate to a medical work. He begs leave therefore, briefly to state, that he thinks Epsom salts dissolved in cold or warm water, more efficacious in curing Cholera than any other one remedy, and has saved many patients with it. But the quantity must be much greater than usually employed for other diseases, or it will not prove efficacious. His Royal Highness' adopted son, Pheng Napoleon, has saved many from the Cholera in this way. The readers who desire to cure with Epsom salts must ask Mr. P. Napoleon for the manner of its use or employment, if they do not know.

His Royal Highness would also recommend the free use of the Jack fruit, even the dried fruit as food during the Cholera, from his own experience and observation of its effect in preventing the symptoms of Cholera. Frequent smelling of camphor, or scenting the room with it, and fumigation by burning Gum Benjamin is also beneficial.

